# Vibrio cholerae CsrA Directly Regulates *varA* To Increase Expression of the Three Nonredundant Csr Small RNAs

**DOI:** 10.1128/mBio.01042-19

**Published:** 2019-06-04

**Authors:** Heidi A. Butz, Alexandra R. Mey, Ashley L. Ciosek, Shelley M. Payne

**Affiliations:** a Department of Molecular Biosciences, The University of Texas at Austin, Austin, Texas, USA; University of Hawaii at Manoa

**Keywords:** CsrA, CsrB, ToxR, VarA, *Vibrio cholerae*, sRNA

## Abstract

CsrA, an RNA-binding global regulator, is an essential protein in Vibrio cholerae. V. cholerae CsrA is regulated by three small RNAs (sRNAs), namely, CsrB, CsrC, and CsrD, which act to sequester and antagonize the activity of CsrA. Although the sRNAs were considered to be largely redundant, we found that they differ in expression, half-life, and the ability to regulate CsrA. Further, we identified a feedback loop in the Csr system in which CsrA increases the synthesis of these antagonistic sRNAs. Because the Csr sRNAs are positively regulated by VarA, we determined the effects of CsrA on VarA levels. The level of VarA was reduced in a *csrA* mutant, and we found that CsrA directly bound to *varA* mRNA in an electrophoretic mobility shift assay *in vitro* and in an CsrA-RNA immunoprecipitation assay *in vivo*. Thus, *varA* mRNA is an *in vivo*-verified direct target of CsrA in V. cholerae, and this is the first demonstration of CsrA directly binding to a *varA*/*uvrY*/*gacA* homolog. Additionally, we demonstrated that a *varA* translational fusion was less active in a *csrA* mutant than in wild-type V. cholerae, suggesting that CsrA enhances *varA* translation. We propose that this autoregulatory feedback loop, in which CsrA increases the production of the nonredundant Csr sRNAs by regulating the amount of VarA, provides a mechanism for fine-tuning the availability of CsrA and, thus, of its downstream targets.

## Abstract (precis)

Vibrio cholerae is a major human pathogen, causing epidemics and pandemics of cholera. V. cholerae persists in the aquatic environment, providing a constant source for human infection. Success in transitioning from the environment to the human host and back requires the bacterium to rapidly respond and to adjust its gene expression and metabolism to these two very different habitats. Our findings show that CsrA, an RNA-binding regulatory protein, plays a central role in regulating these transitions. CsrA activity is controlled by the antagonistic sRNAs CsrB, CsrC, and CsrD, and these sRNAs respond to changes in the availability of nutrients. CsrA autoregulates its own activity by controlling these sRNAs via their primary regulator VarA. Thus, the change in CsrA availability in response to nutrient availability allows V. cholerae to alter gene expression in response to environmental cues.

## INTRODUCTION

The Gram-negative bacterium Vibrio cholerae causes the life-threatening diarrheal disease cholera. It is endemic in multiple developing countries and is associated with epidemics and pandemics. In the ongoing V. cholerae epidemic in Yemen, as many as 10,000 new cases a week are being reported currently (1). V. cholerae is a natural inhabitant of aqueous environments, and in developing countries with poor water sanitation capabilities, it can contaminate the water supply.

The successful transition of V. cholerae between the aqueous and human intestinal environments depends in part on global regulatory proteins. In response to environmental cues, global regulators act to rapidly alter the expression of a large number of downstream targets to orchestrate a response that promotes adaptation and survival. One such global regulator in V. cholerae is the RNA-binding protein CsrA, which has been shown to link extracellular input with pathways required for pathogenesis in the host. In V. cholerae, CsrA regulates quorum sensing, which controls biofilm formation and virulence gene expression (2), and CsrA plays a critical role in the synthesis of a major virulence factor, ToxR, in response to specific nutrients (3). Not surprisingly, CsrA was shown to be critical for the survival of V. cholerae in a mouse model of V. cholerae infection (4).

CsrA is a small homodimeric protein with two identical RNA-binding surfaces, enabling one CsrA dimer to bind to two locations on the target RNA (5–7). To regulate expression, CsrA typically binds to the 5′ untranslated region (5′ UTR) of mRNAs in a manner that either positively or negatively affects transcript stability (8, 9), transcription termination (10), and/or efficiency of translation (11–16).

**FIG 1:**
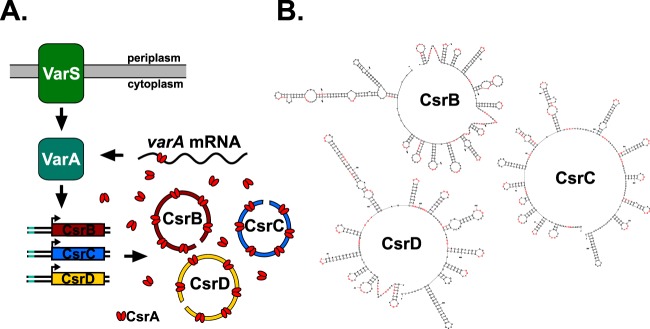
Csr system in V. cholerae. (A) The two-component system VarS and VarA regulates the activity of CsrA. The inner membrane protein VarS is a sensor kinase that activates VarA via phosphorylation. Activated VarA binds to the promoters of three small RNAs, namely, CsrB, CsrC, and CsrD (CsrB/C/D, or the Csr sRNAs), to induce their transcription; the portion of the Csr sRNA promoters indicated in green represents the VarA recognition sequence. When expressed, CsrB/C/D act to sequester CsrA, antagonizing the activity of CsrA. The data determined in the present study indicate that CsrA directly binds to *varA* mRNA to positively regulate protein expression, introducing a regulatory feedback loop in the Var/Csr pathway in V. cholerae. (B) The RNA folding program mFold was used to generate the secondary structures of CsrB/C/D. The GGA CsrA-binding sites are highlighted by the red lettering.

In V. cholerae, the activity of CsrA is regulated by the two-component system (TCS) VarS/VarA (2, 17), outlined in Fig. 1A. This system is homologous to BarA/UvrY in Escherichia coli, GacS/GacA in *Pseudomonas* species, and BarA/SirA in *Salmonella* species. In E. coli, BarA is an inner membrane tripartite sensor kinase that has been shown to directly activate its cognate transcriptional activator UvrY via transphosphorylation (18). Once activated, phospho-UvrY binds to the promoters of the small RNAs (sRNAs) CsrB and CsrC to induce their transcription (18–20). BarA and UvrY share 53% and 74% amino acid identity with VarS and VarA, respectively, suggesting that VarS and VarA function in a similar manner in V. cholerae. However, in contrast to UvrY in E. coli, which induces the expression of two sRNAs, VarA induces the transcription of the three sRNAs CsrB, CsrC, and CsrD in V. cholerae (2, 21).

In E. coli, the CsrB sRNA has been shown to bind directly to CsrA and antagonize its activity (22). The Csr sRNAs have highly ordered secondary structures that contain multiple CsrA-binding sites, allowing the sRNA to sequester CsrA, thus preventing CsrA from actively regulating its RNA targets (23, 24). The optimal CsrA-binding site was determined to be a GGA motif located within the loop region of an RNA stem-loop (25). In V. cholerae, CsrB has 28 GGA motifs whereas CsrC and CsrD have 23 GGA motifs each. Interestingly, the predicted secondary structures of CsrB, CsrC, and CsrD all contain 11 GGA motifs located in loop regions (Fig. 1B) (26), suggesting that the three sRNAs potentially sequester similar amounts of CsrA. Since the Csr sRNAs have similar secondary structures and have been reported to exhibit similar expression profiles (2, 27), it has been suggested that they function in a redundant manner to regulate CsrA activity. As the amount of CsrA within a cell typically does not change, altering the expression of the Csr sRNAs allows rapid regulation of CsrA activity by controlling its availability (28).

Both extrinsic and intrinsic signals control the expression of the Csr sRNAs. In E. coli, Csr sRNA expression is influenced by glucose metabolic end products, amino acids, and pH (27, 29, 30). Intrinsic regulatory feedback loops have been described in the Csr system in both E. coli and *Pseudomonas* species (31–35). For example, CsrA has been shown to promote the expression of the Csr sRNAs (32, 34, 36, 37). Although the exact mechanism remains unclear, CsrA is believed to indirectly promote BarA kinase activity and UvrY protein synthesis (20, 37). This feedback mechanism, whereby CsrA positively regulates the expression of its antagonistic sRNAs, likely serves to prevent drastic oscillations in CsrA activity. The Csr, BarA, and UvrY system has been extensively studied in E. coli (reviewed in references 38–40); however, the regulation of this system in V. cholerae is not well understood.

In the present study, we showed that, in V. cholerae, the Csr sRNAs are differentially regulated and are not completely redundant in their ability to regulate CsrA. Additionally, in response to nutrient supplementation, the pool of Csr sRNAs decreases, likely shifting the equilibrium from sequestered CsrA toward available CsrA. This increase in the level of active CsrA, a global regulator, could play an important role in how V. cholerae successfully senses and responds to environmental changes. We also uncovered an autoregulatory loop in which CsrA controls the production of its antagonistic sRNAs. We showed that CsrA positively regulates VarA protein levels, ultimately leading to increased transcription of the *csr* sRNA genes. Further, we demonstrated direct binding of CsrA to the *varA* transcript, both *in vitro* and *in vivo*. Our data are consistent with CsrA directly regulating *varA* by binding the *varA* mRNA and increasing its translation. Importantly, *varA* is a verified direct mRNA target of CsrA in V. cholerae *in vivo*, demonstrating a direct role for CsrA in the regulation of a UvrY/GacA/SirA homolog.

## RESULTS

### NRES affects the expression of the *csr* sRNAs but not the amount of CsrA.

**FIG 2:**
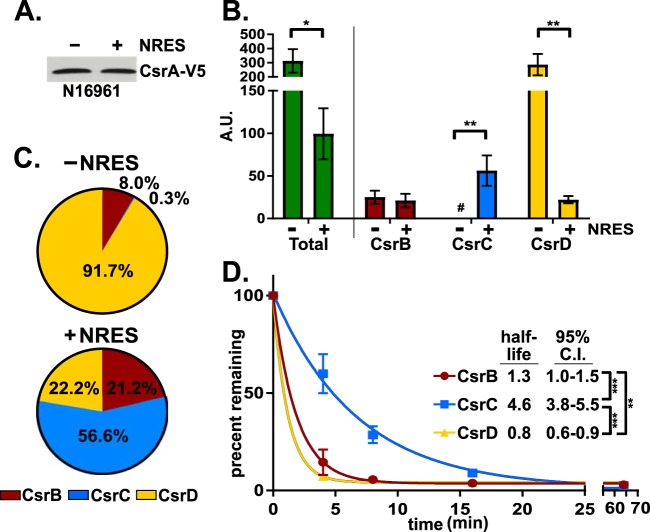
The Csr sRNA levels, but not the CsrA protein levels, change in response to NRES. (A) The wild-type strain, N16961, harboring a V5 epitope tagged CsrA, was grown in minimal medium with and without NRES, and whole-cell proteins were resolved and immunoblotted with anti-V5 antiserum. (B) The total abundance, shown in arbitrary units (A.U.), of each Csr sRNA was determined by the use of standard curves. #, <1 A.U. of CsrC in minimal medium. The *P* values were determined by an unpaired, two-tailed Student's *t* test from the A.U. values (*, *P* < 0.05; **, *P* < 0.01). The normalized means and standard deviations (error bars) were calculated from three biological replicates. (C) Relative proportions of CsrB/C/D present when the wild-type strain was grown in the absence and presence of NRES. The amount of Csr sRNA present in the experiment whose results are presented in panel B was converted to a percentage. (D) The rate of decay for CsrB, CsrC, and CsrD was measured by qRT-PCR from wild-type cells grown to mid-log phase in minimal medium supplemented with NRES. Rifampin was added to stop transcription, and cells were harvested immediately after rifampin addition (*t* = 0) and at 4, 8, 16, and 64 min after rifampin addition. The half-lives, along with the 95% confidence intervals (C.I.), were determined using one-phase decay analysis (a nonlinear regression method) and GraphPad Prism 7 software. The extra sum-of-squares F test was used to compare the decay rate constants determined for the sample sets (*, *P* < 0.05; **, *P* < 0.01; ***, *P* < 0.001).

Our previous work demonstrated a role for CsrA in promoting ToxR production in response to specific amino acids in the growth medium (3). When wild-type V. cholerae is grown in a minimal medium, ToxR protein levels are low; however, under conditions of growth in a minimal medium supplemented with the four amino acids asparagine, arginine, glutamic acid, and serine (NRES), ToxR levels increase (3), and this increase in ToxR is positively regulated by CsrA (4). The CsrA-dependent increase in ToxR protein levels could reflect either an increase in the total amount of CsrA or an increase in the proportion of CsrA that is available. To determine the level of CsrA in response to NRES, a construct containing a V5 epitope-tagged CsrA expressed from its native promoter was introduced into the wild-type strain. The V5 tag did not interfere with the ability of CsrA to regulate ToxR levels (see Fig. S1 in the supplemental material). The total amount of CsrA-V5 protein was the same whether the strain was grown in the absence or in the presence of NRES, indicating that the amount of CsrA was not affected by NRES (Fig. 2A). Since the absolute level of CsrA did not change, it is likely that the differences in CsrA-mediated regulation seen under the two conditions reflected differences in the amounts of active CsrA. To test whether NRES influences the level of the CsrA-antagonistic Csr sRNAs and thus the level of free, active CsrA, we quantified the amounts of CsrB, CsrC, and CsrD present when V. cholerae was grown in the absence or presence of NRES. We found that the total amount of the Csr sRNAs was 3-fold higher in the absence of NRES (Fig. 2B). These data suggest that, in the absence of NRES, the total amount of CsrB/C/D is high, resulting in increased sequestration of CsrA. In the presence of NRES, the total amount of CsrB/C/D is lower, leading to smaller amounts of sequestered CsrA and therefore higher amounts of available CsrA.

10.1128/mBio.01042-19.1 FIG S1 V5-tagged CsrA restores ToxR expression in N*csrA*.R6H. N16961, N*csrA*.R6H, N*csrA*.R6H/pF*csrA*, and N*csrA*.R6H/pF*csrA*-V5 were grown in minimal medium with and without NRES. Cells were harvested at mid-log phase and resuspended in LB sample buffer, and whole-cell proteins were resolved by 10% polyacrylamide SDS-PAGE. The gels were processed and immunoblotted with anti-ToxR antiserum. Download FIG S1, EPS file, 0.1 MB. Copyright © 2019 Butz et al. 2019 Butz et al. This content is distributed under the terms of the Creative Commons Attribution 4.0 International license.

### The V. cholerae *csr* sRNAs have different intrinsic properties.

Although the total amounts of Csr sRNAs differed when V. cholerae was grown with or without NRES, it was unclear what the contribution of each individual sRNA was to the total amount observed under each condition. When the Csr sRNAs were assessed individually, we found that they differed significantly in abundance under the two conditions. The level of CsrC increased 67-fold in the presence of NRES, whereas that of CsrD decreased 12-fold, and CsrB was unaffected by the addition of the amino acids (Fig. 2B). For consistency with other experiments, random primers were used to generate the cDNA analyzed in the experiment presented in Fig. 2B. To verify that the observed trends were not affected by differences in the efficiency of priming by the random primers during cDNA synthesis, the experiment was repeated with a reverse transcriptase (RT) primer that had 100% homology to the same 3′ region in each of the Csr sRNAs (Fig. S2A). In addition, equivalent efficiencies of the PCR step in this experiment were verified by generating standard curves (Fig. S2B). Although the relative proportions of the Csr sRNAs differed slightly between these two approaches, the overall trends were consistent. From these data, it also became clear that the relative proportions of the Csr sRNAs were starkly different in the absence and presence of NRES (Fig. 2C). In the absence of NRES, CsrD was the major Csr sRNA present, followed by CsrB, while CsrC represented <1% of the total Csr sRNA. In contrast, in the presence of NRES, CsrC represented a substantial portion of the total Csr sRNA.

10.1128/mBio.01042-19.2 FIG S2 Verification of priming efficiency in RT and qPCR. (A) Total abundance of each Csr sRNA shown in raw nanograms (ng). To ensure that the RT priming efficiencies were the same for all three sRNAs, a specific probe that binds to identical sequences in the 3′ end of all three sRNAs was used. The abundance of each was determined by standard curves. The *P* values were determined by an unpaired, two-tailed Student’s *t* test using the raw nanogram values (**, *P* < 0.01; *** *P* < 0.001). The means and standard deviations (error bars) were calculated from three biological replicates. (B) Priming efficiency for qPCR analysis of the cDNA as described in the panel A legend. The slope, *R*^2^ value, and percentage of efficiency determined for each of the primer sets, which were extrapolated from their respective standard curves, are shown. Download FIG S2, EPS file, 0.10 MB. Copyright © 2019 Butz et al. 2019 Butz et al. This content is distributed under the terms of the Creative Commons Attribution 4.0 International license.

We also examined the rate of decay of CsrB/C/D in the wild-type strain grown in minimal medium supplemented with NRES. The Csr sRNAs have significantly different half-lives, with CsrB having a half-life of 1.3 min, CsrD a half-life of 0.8 min, and CsrC a half-life of 4.6 min (Fig. 2D). As studies of Csr regulation in other species showed the Csr sRNAs to be regulated similarly to each other (2, 32), the finding that the V. cholerae Csr sRNAs have different intrinsic properties was unexpected. These data may indicate that the Csr sRNAs differ in their abilities to regulate CsrA activity.

### CsrC is not functionally redundant with CsrB or CsrD.

To determine whether the Csr sRNAs have different or redundant functions, strains were generated containing only one Csr sRNA, under the control of its native promoter, in their genome. These strains were named N*csrB-only*, N*csrC-only*, and N*csrD-only*. Strains with extremely high levels of active CsrA often acquire suppressor mutations in *csrA* (4, 41); therefore, we confirmed by sequencing that the N*csrB-only*, N*csrC-only*, and N*csrD-only* strains each carried the wild-type *csrA* allele.

**FIG 3:**
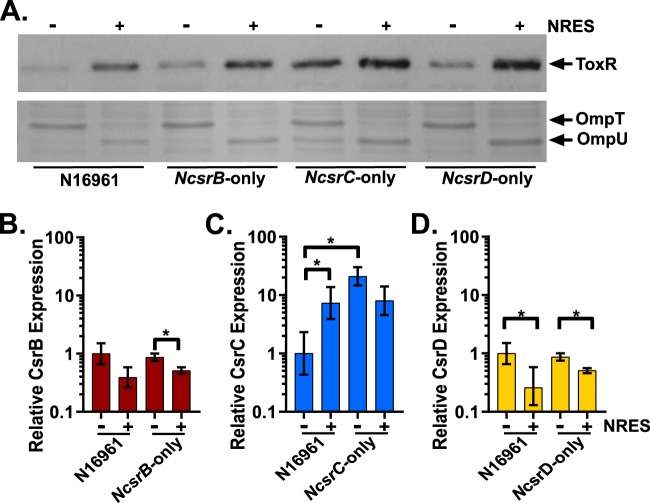
N*csrC*-*only* has increased CsrA activity in minimal medium. (A) N16961, N*csrB*-*only*, N*csrC*-*only*, and N*csrD*-*only* were grown in minimal medium with and without NRES. Whole-cell proteins were resolved and immunoblotted with anti-ToxR antiserum (top panel) or stained with Coomassie blue (bottom panel). The proteins in both the immunoblot and Coomassie-stained gel were from the same samples and are representative of results from at least three biological replicates. (B to D) CsrB (B), CsrC (C), and CsrD (D) expression levels were examined by qRT-PCR in strain N16961 and strains N*csrB*-*only*, N*csrC*-*only*, and N*csrD*-*only*, respectively. All strains were grown as described for panel A. The levels of CsrB/C/D were normalized to the internal control *secA* level. In each panel, the wild-type (WT) level seen in the absence of NRES is set to a value of 1. The *P* values were determined by an unpaired, two-tailed Student's *t* test from the Δ*C_T_* values (*, *P* < 0.05), comparing N16961 to the indicated mutant and comparing the levels of growth in minimal medium with or without NRES. Differences that are statistically significant are indicated on the figures. The relative means and standard deviations (error bars) were calculated from three biological replicates.

To test if the Csr sRNAs were redundant with respect to their ability to regulate CsrA, the amount of ToxR protein was examined from cultures grown in minimal medium with and without NRES. Both the N*csrB-only* and N*csrD-only* strains exhibited wild-type regulation of ToxR, with low ToxR levels seen in the absence and high levels in the presence of NRES. In contrast, the N*csrC*-*only* strain produced high amounts of ToxR under both conditions, suggesting a high level of active CsrA, even in the absence of NRES (Fig. 3A). To confirm the relevance of the observed increase in ToxR, the levels of two ToxR-regulated targets, the outer membrane porin proteins OmpT and OmpU, were determined as well. ToxR inversely regulates expression of the porin genes by repressing *ompT* and inducing *ompU*; thus, in a minimal medium, when ToxR levels are low, predominantly OmpT is produced, and in NRES, when ToxR levels are high, OmpU is the dominant porin (3). As expected, based on the ToxR results described above, both the N*csrB-only* and N*csrD-only* strains exhibited the wild-type Omp profile; however, the N*csrC*-*only* strain produced OmpU in the absence of NRES (Fig. 3A), consistent with a higher than normal level of ToxR in this strain under conditions of growth without NRES. The increase in ToxR levels in the N*csrC*-*only* strain was likely due to high levels of active CsrA in the absence of the other two Csr sRNAs. The explanation for this result could be either that the total amount of CsrC under this condition was not high enough to effectively control the level of active CsrA or that CsrC is not as efficient as CsrB and CsrD in sequestering active CsrA.

To determine whether the *NcsrC*-*only* strain is unable to regulate CsrA in the absence of NRES because of low levels of the sRNA, the amounts of the Csr sRNAs produced in the single-sRNA-expressing strains were examined in the absence and presence of NRES. Neither the CsrB levels nor the CsrD levels changed in the N*csrB-only* strain (Fig. 3B) or the N*csrD-only* strain (Fig. 3D), respectively, in comparison to the wild type. However, the levels of CsrC increased significantly in the N*csrC-only* strain compared to wild type (Fig. 3C). This suggests that loss of CsrB and CsrD resulted in a compensatory increase in CsrC. Importantly, despite the increase in its amount, CsrC alone was unable to efficiently sequester CsrA, resulting in high levels of ToxR (Fig. 3A). Together, these data demonstrate that the V. cholerae Csr sRNAs are not completely redundant and that differences in regulation, stability, and function of these sRNAs likely contribute to the complexity of this pathway.

### CsrA controls the level of the csr sRNAs.

**FIG 4:**
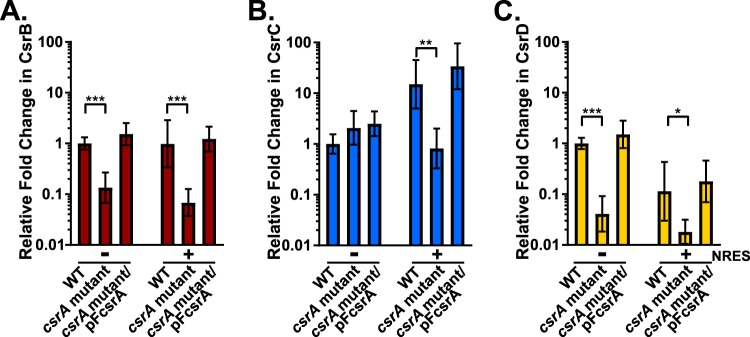
CsrA positively affects the expression of the *csr* sRNAs. CsrB (A), CsrC (B), and CsrD (C) expression levels were examined by qRT-PCR in N16961 (WT), N*csrA*.R6H (*csrA* mutant), and the complemented strain N*csrA*.R6H/pF*csrA* (*csrA* mutant/pF*csrA*). All strains were grown in minimal medium with and without NRES. The levels of CsrB/C/D were normalized to the internal control *secA* level. In each panel, the WT level seen in the absence of NRES is set to a value of 1. The *P* values were determined by an unpaired, two-tailed Student's *t* test from the Δ*C_T_* values (*, *P* < 0.05; **, *P* < 0.01; ***, *P* < 0.001). The relative means and standard deviations (error bars) were calculated from four biological replicates.

Our previous work indicated that V. cholerae must maintain the level of active CsrA within an appropriate range to prevent deleterious effects; a complete deletion of the *csrA* gene was lethal, and overexpression of *csrA* caused significant growth defects as well (4). One way to maintain the appropriate level of CsrA would be to regulate the level of Csr sRNAs produced in response to the level of available CsrA. To test if CsrA itself plays a role in regulating the Csr sRNAs, we measured sRNA levels in strain N*csrA*.R6H, which carries a single amino acid substitution in CsrA that eliminates its ability to regulate ToxR in response to NRES but retains sufficient activity for cell viability (4). The levels of CsrB, CsrC, and CsrD were determined by reverse transcriptase quantitative PCR (qRT-PCR) in the wild-type and N*csrA*.R6H strains grown in minimal medium with and without NRES. The levels of all three sRNAs were significantly reduced in N*csrA*.R6H in the presence of NRES (Fig. 4). In addition, CsrB and CsrD levels were reduced in the *csrA* mutant in the minimal medium without NRES. In the absence of NRES, the level of CsrC did not decrease significantly in the *csrA* mutant, but this was likely because the level of CsrC was already low under this condition (Fig. 2C). Wild-type levels of the Csr sRNAs in N*csrA*.R6H were restored by supplying the wild-type *csrA* allele on a plasmid (pF*csrA*), suggesting that the observed sRNA decrease was due to the *csrA* mutation. This suggests a general requirement for CsrA activity in controlling the level of the Csr sRNAs. Possible mechanisms of regulation by CsrA include regulation of sRNA stability, possibly through direct interactions with the sRNAs, or regulation of sRNA synthesis through control of components in the pathway that lead to sRNA transcription.

### CsrA positively affects the transcription of *csrB*, *csrC*, and *csrD*.

**FIG 5:**
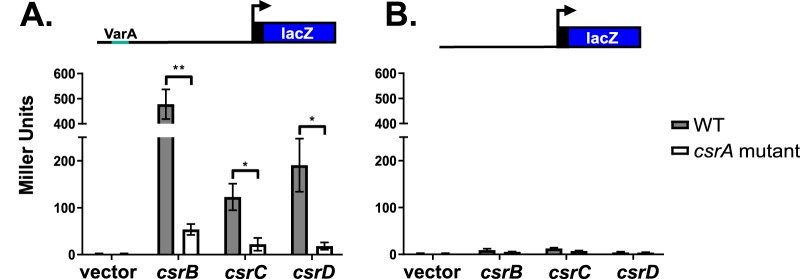
CsrA positively affects the transcription of the *csr* sRNA genes. The activities of the full-length promoter fusions (A) and the truncated promoter fusions (B) for CsrB, CsrC, and CsrD were measured in the N*lacZ*::*kan* (WT) and N*csrA.R6H.lacZ*::*kan* (*csrA* mutant) strains. The truncated fusions lack 50 nucleotides on the 5′ end encompassing the VarA-binding site, shown as the light blue segment in the schematic. The means are plotted, and the error bars represent standard deviations of data from three biological replicates. The *P* values were calculated using the unpaired *t* test (*, *P* < 0.05; **, *P* < 0.01).

To determine whether CsrA activity influences the transcription of the *csr* sRNA genes, a series of transcriptional reporters containing either the full-length promoter or a truncated fragment were constructed. The full-length promoter extends from 20 nucleotides upstream of the predicted VarA-binding site, TGTGCGAGATCTCTTACA (2), to 3 nucleotides into the transcribed region of the *csr* sRNA gene (represented in the schematic in Fig. 5A). If the reduction in the levels of the Csr sRNAs in N*csrA*.R6H was in part due to CsrA regulating the transcription of the *csr* sRNA genes, the transcriptional reporters should produce less β-galactosidase in N*csrA*.R6H than in the wild type. To measure the levels of β-galactosidase produced from the reporters, the chromosomal *lacZ* gene was disrupted in both strains, generating N*lacZ*::*kan* and N*csrA*.R6H.*lacZ*::*kan*. These strains harboring the reporters were grown in minimal medium supplemented with NRES. Analysis of the results seen with the full-length reporters showed that the level of transcription for each of the Csr sRNAs was reduced in the *csrA* mutant in comparison to the wild-type strain (Fig. 5A). Transcription of *csrB* was reduced by ∼9-fold, *csrC* transcription was reduced by ∼6-fold, and *csrD* transcription was reduced by ∼11-fold. These results suggest that CsrA positively regulates the Csr sRNAs at the level of transcription. The mechanism of CsrA-mediated transcriptional regulation of CsrB, CsrC, and CsrD is likely indirect, since CsrA is not known to bind directly to DNA to affect the rate of transcription (36).

### Transcription of *csrB*, *csrC*, and *csrD* requires VarA binding promoter motifs.

A potential candidate for a direct CsrA regulatory target in the Csr pathway is VarA (Fig. 1A). VarA is required for the expression of all three Csr sRNAs, and the promoter regions of the sRNA genes all contain a predicted VarA-binding site (2). To verify the importance of the predicted VarA-binding sites for sRNA transcription, we measured expression from a truncated fusion lacking 50 nucleotides on the 5′ end that included the VarA recognition sequence (represented in the schematic in Fig. 5B). There was minimal expression detected from the truncated reporters in the wild-type strain and in the *csrA* mutant (Fig. 5B), showing that the predicted VarA recognition sequence is required for transcription of the *csr* sRNA genes. This is consistent with findings in Pseudomonas fluorescens where the GacA-binding site was shown previously to be required for transcription of the sRNAs (42), but this had not been demonstrated previously in V. cholerae. Taken together, these data suggest that, since both CsrA and VarA play a role in the transcription of the *csr* sRNAs, there may be some interplay between these two key regulatory factors in the Csr pathway.

### There is less VarA protein produced in N*csrA*.R6H than in the wild type.

**FIG 6:**
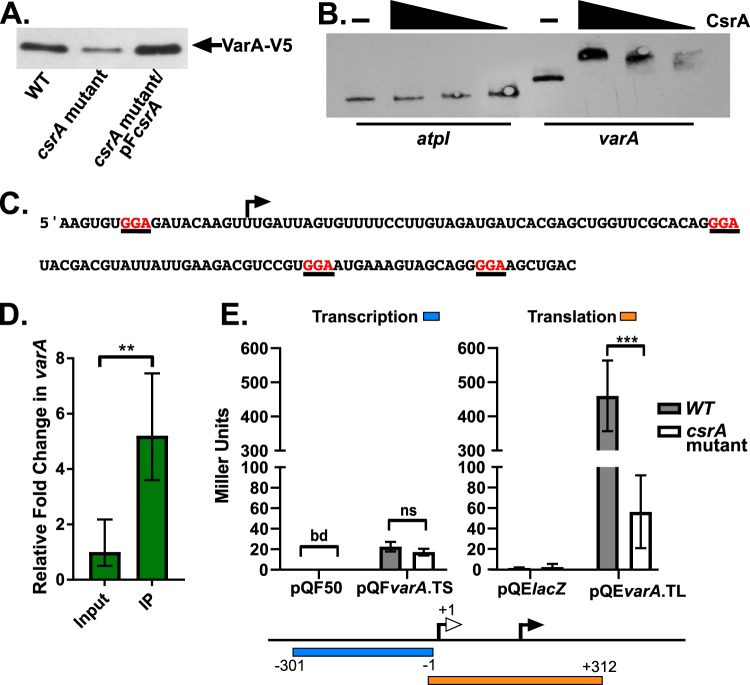
CsrA positively affects the level of VarA and binds directly to the *varA* mRNA. (A) N*varA*-V5 (WT), N*csrA*.R6H.*varA*-V5 (*csrA* mutant), and N*csrA*.R6H.*varA*-V5/pF*csrA* (*csrA* mutant/pF*csrA*) were grown in minimal medium supplemented with NRES. Cells were harvested at mid-log phase, and whole-cell proteins were resolved and immunoblotted with anti-V5 antiserum. The immunoblot is representative of results from at least three biological replicates. (B) The RNA EMSA was performed with purified CsrA, the negative-control 5′ biotinylated *atpI* RNA, and the 5′ biotinylated *varA* RNA. The highest concentration of CsrA in the gradient was 40 nM, followed by 8 nM and 1.6 nM. (C) The sequence of the *varA* oligonucleotide used for the RNA EMSA whose results are presented in panel B. The closed arrow is positioned above the annotated start of translation UUG (61), and GGA motifs are indicated in red and underlined. (D) *In vivo* CsrA-RNA immunoprecipitation showing that the *varA* transcript was enriched ∼5-fold in the immunoprecipitated (IP) sample compared to the input RNA population. The *P* values were determined by an unpaired, two-tailed Student's *t* test from the Δ*C_T_* values (**, *P* < 0.01). (E) The *varA* transcriptional reporter, pQF*varA*.TS, includes the *varA* promoter sequence (−301 to −1 relative to the transcriptional start site, blue bar) fused to *lacZ*, while the *varA* translational fusion, pQE*varA*.TL, includes the 5′ UTR and 73 amino acids of the coding sequence (−1 to +312 relative to the start of transcription, orange bar) as indicated in the schematic below the panel. The translational fusion uses the plasmid T5 promoter. The open arrow represents the start of transcription, while the closed arrow indicates the start of translation. The activity of these reporters was measured in the N*lacZ*::*kan* (WT) and N*csrA*.R6H*.lacZ*::*kan* (*csrA* mutant) strains. IPTG (100 μM) was present in the growth medium during testing of the translational reporter. To generate the plotted means and standard deviations (error bars), three biological replicates were used for the transcriptional reporter, and four biological replicates were used for the translational reporter. The *P* values were calculated using the unpaired, two-tailed Student's *t* test (ns, not significant; ***, *P* < 0.001; bd, below detection).

To determine whether CsrA affects the transcription of the Csr sRNA genes by controlling the expression of VarA, we measured the amount of VarA protein in the wild-type strain and the *csrA* mutant. To visualize the VarA protein, allelic exchange was used to replace the wild-type *varA* allele with an allele encoding a VarA protein with the V5 epitope tag fused to the carboxyl terminus. This approach was performed in both the wild-type strain and N*csrA*.R6H, generating the strains N*varA*-V5 and N*csrA*.R6H.*varA-*V5. The levels of VarA were determined by Western blotting in the wild-type strain, the *csrA* mutant, and the complemented strain (Fig. 6A). There was less VarA seen with the *csrA* mutant than with the wild-type strain, and this was found to be restored by ectopically supplying *csrA*, indicating that the decrease in VarA protein levels in N*csrA*.R6H was due to the *csrA* mutation. Thus, in V. cholerae, CsrA positively regulates VarA protein levels.

### CsrA binds directly to *varA* mRNA *in vitro*.

To examine if CsrA binds directly to *varA* mRNA, we performed an *in vitro* RNA electrophoretic mobility shift assay (REMSA) with purified CsrA and a biotinylated *varA* RNA oligonucleotide. The *varA* oligonucleotide begins 18 nucleotides before the start of translation and includes 102 nucleotides of the predicted coding region. This segment of the *varA* transcript contains 4 GGA motifs that could act as potential CsrA-binding sites (Fig. 6C). The REMSA showed binding of the *varA* oligonucleotide at 1.6 nM CsrA (Fig. 6B). As the concentration of CsrA increased in 5-fold increments, the amount of the shifted *varA* species also increased. There was no change in the mobility of CsrA in the presence of a nonspecific RNA oligonucleotide, *atpI*, which is not a predicted CsrA target, suggesting that CsrA binding to *varA* mRNA *in vitro* is specific (Fig. 6B).

### CsrA directly binds to *varA* mRNA *in vivo*.

To confirm that CsrA binds to *varA* mRNA *in vivo*, we performed an *in vivo* CsrA-RNA coimmunoprecipitation assay using a V5-tagged CsrA expressed from an arabinose-inducible vector. N*csrA*.R6H carrying the CsrA-V5 construct was grown to mid-log phase, and *csrA*-V5 expression was induced. The cells were lysed, 5% of the initial lysate was removed, and the RNA was isolated. The CsrA-RNA complexes were isolated from the remaining lysate using beads coated with anti-V5 antibodies. To determine enrichment, the relative amount of RNA that coimmunoprecipitated with CsrA was compared to the initial input population of RNA. It was determined by qRT-PCR that the level of *varA* mRNA was significantly enriched (5.2-fold, *P* = 0.002) in the CsrA-RNA coimmunoprecipitated sample (Fig. 6D). This suggests that CsrA directly binds to the *varA* mRNA *in vivo*.

Binding of CsrA to the *varA* transcript could increase VarA levels by affecting the efficiency of its translation. A translational *varA*::*lacZ* fusion, as well as a transcriptional fusion for use as a control for any indirect effects of CsrA on transcription, were constructed (Fig. 6E). The levels of β-galactosidase from the transcriptional fusion were the same in the wild-type and *csrA* mutant strains. In contrast, the translational fusion showed a significant decrease in the level of β-galactosidase in the *csrA* mutant. These data support a role for CsrA in the translation, but not transcription, of *varA*.

## DISCUSSION

In order to transition efficiently between its native aquatic environment and the human host, V. cholerae must sense changes in its environment and respond by rapidly altering gene expression to promote adaptation. In V. cholerae, sensing through the two-component system VarS/VarA has been shown to be required both for colonization of the mammalian host (43) and for the bacterium to successfully transition from the host back into the aqueous environment (41). The VarS/VarA TCS induces the transcription of the Csr sRNAs, CsrB/C/D, which act to sequester the global regulator, CsrA. Since VarS/VarA play a critical role in transitioning between environments, and because a major cue of a new environment could be changes in the nutritional status, we hypothesized that the Csr system in V. cholerae responds to the presence of specific nutrients in its environment. In support of this hypothesis, nutrient availability has been linked to regulation of the Csr system in multiple bacterial species. In E. coli, the levels of CsrB and CsrC are influenced by glucose uptake and metabolism through effects on sRNA synthesis and turnover (29, 44, 45). The amino acid starvation response also plays a role in maintaining Csr sRNA levels: CsrB and CsrC were more highly expressed during growth under nutrient-limiting conditions, and the addition of amino acids to the medium had a repressive effect on Csr sRNA production (27, 29). Regulation of Csr sRNA production in response to nutrient conditions was also observed in *Yersinia* and *Shewanella* species (46, 47). In this study, we showed that the total amount of the Csr sRNAs in V. cholerae decreased 3-fold in response to amino acid (NRES) supplementation. Importantly, this decrease in the total pool of the Csr sRNAs likely shifted the equilibrium from a greater amount of sequestered CsrA to a greater amount of active CsrA. This shift in the equilibrium toward a greater amount of active CsrA in response to the nutrient status may contribute to some of the changes in gene expression that are critical for V. cholerae to successfully adapt to a new environment. For example, in low-nutrient aquatic environments, it would be advantageous for V. cholerae to express outer membrane porins with greater permeability with respect to potential nutrients; however, within the human host, where the nutrient level is high, porins with increased permeability are no longer necessary and may indeed become a liability as the bacteria encounter host-derived toxic compounds that can enter the cell through permissive porins. Thus, a system is needed to control the outer membrane porin composition in response to specific environmental signals. We showed previously that V. cholerae uses the Csr system to link nutritional status to porin synthesis: CsrA promotes an increase in ToxR levels in response to amino acids (NRES), leading to production of OmpU, the porin with greater barrier function. Under nutrient-limiting conditions with no NRES, the CsrA-mediated increase in ToxR does not occur, and the more permissive porin OmpT is produced (4). In this way, the Csr system acts as a sensor of nutrient conditions to ensure appropriate porin production under each condition.

The presence of multiple Csr sRNAs in V. cholerae suggested that they might have separate functions. Lenz et al. (2) constructed strains with only one of the Csr sRNAs and found that all three single sRNA strains were identical to the wild type with respect to quorum sensing, suggesting that CsrB, CsrC, and CsrD are redundant; however, our data show that they are not fully redundant. Under low-nutrient conditions, i.e., during growth in minimal medium without NRES, CsrC, unlike CsrB and CsrD, was unable to efficiently regulate CsrA. CsrC also differed from CsrB and CsrD in that it was more stable, and CsrC showed a distinct pattern of regulation in response to NRES. Thus, the Csr sRNAs have distinct functions as well as overlapping functions. Studying the functions of each of the three Csr sRNAs under additional environmental conditions will likely reveal further differences in their functions and roles with respect to each of the three sRNAs.

Comparative analyses of the sequences of the sRNAs provided additional insights into differences among the sRNAs. We performed a cluster alignment of the primary sequences of V. cholerae CsrB/C/D along with Csr sRNAs found in E. coli, Shigella flexneri, Salmonella enterica, *Yersinia* species, and *Pseudomonas* species (see Fig. S3 in the supplemental material). As reported by Lenz et al. (2), the V. cholerae Csr sRNAs are more similar to each other than to those of any of the other species examined. This suggests that the three Csr sRNAs arose through gene duplication events after V. cholerae diverged from the other species. Interestingly, we noted that, in V. cholerae, CsrB and CsrD are more similar to each other than either is to CsrC, suggesting that CsrC may have diverged in function as well as sequence. Determination of homologs of CsrB, CsrC, and CsrD among members of the *Vibrionaceae* family revealed that both CsrB and CsrD are found throughout the family, whereas CsrC is found only in V. cholerae strains. The fact that CsrC is unique to V. cholerae and is not fully redundant with CsrB and CsrD suggests that CsrC may have a unique role in regulating CsrA and downstream virulence gene expression in V. cholerae.

10.1128/mBio.01042-19.3 FIG S3 Guide tree of the Csr sRNAs from multiple bacterial species. The DNA sequences of the *csr* sRNA genes from the indicated species were aligned through multiple ClustalW analyses. Download FIG S3, EPS file, 0.1 MB. Copyright © 2019 Butz et al. 2019 Butz et al. This content is distributed under the terms of the Creative Commons Attribution 4.0 International license.

The expression of all three Csr sRNAs decreased significantly in a *csrA* mutant, indicating a feedback mechanism by which CsrA regulates the level of its antagonistic sRNAs in V. cholerae. The presence of such a feedback mechanism is not surprising, given the importance of controlling the level of active CsrA to prevent deleterious effects in the cell. We showed previously that disrupting *varA* caused significant growth defects, most likely due to an overabundance of active CsrA in the absence of the CsrA sRNAs, which depended on VarA for their expression (4). The *varA* mutant rapidly accumulated suppressor mutations in *csrA*, which restored normal growth, presumably by bringing the level of CsrA activity back into an acceptable range. Others have observed comparable suppression of Var and Gac mutant growth phenotypes through spontaneous mutation of *csrA* (41, 48). Further, while a complete deletion of *csrA* is lethal, point mutations and a small C-terminal deletion were possible, resulting in attenuated (but not completely inactive) forms of CsrA (2, 4, 41). Through the feedback loop described here, a mechanism emerges to explain how the cell copes with producing a less active form of CsrA: a less active CsrA, for example, CsrA.R6H, is tolerated if the degree to which it is sequestered by the antagonistic Csr sRNAs remains relatively low. This is accomplished through the inability of CsrA.R6H to stimulate VarA-dependent Csr sRNA expression, resulting in significantly diminished sRNA production.

Negative-feedback regulation, representing the mechanism by which CsrA controls the levels of the Csr sRNAs, has been observed in E. coli and *Salmonella*, although the exact nature of the mechanism remains unclear (34, 36, 37, 49). In E. coli, UvrY protein levels decreased in a *csrA* mutant, but as CsrA binding to the *uvrY* transcript has not been observed and as no CsrA-binding site has been identified *in silico*, the mechanism is predicted to be indirect (20, 37). This is supported by findings in E. coli and *Salmonella* showing that the *varA* homolog transcript did not copurify with CsrA in CsrA-RNA coimmunoprecipitation experiments in either species (50–52). In contrast, we demonstrated here that CsrA binds directly to the *varA* mRNA in V. cholerae, both *in vitro* and *in vivo*. One factor that could promote CsrA binding to the *varA* transcript, but not to *uvrY*/*sirA*, is the presence of additional GGA motifs in the 5′ region of the *varA* transcript. One of the V. cholerae *varA* GGA motifs is nearly identical to the extended CsrA consensus sequence (RUACARGGAUGU) (25). Although the exact mechanism remains to be determined, the data presented in this study are consistent with a mechanism in which CsrA binding to the *varA* transcript increases its efficiency of translation, ultimately resulting in an increase in VarA levels. The proposed effect on translation is supported by the observation that there was significantly less β-galactosidase produced from a *varA*::*lacZ* translational fusion in the *csrA* mutant than in the parental, CsrA-positive strain.

In this study, we showed that, in V. cholerae, CsrA positively regulates the expression of the Csr sRNAs through its effects on VarA. We found that CsrA positively regulates VarA protein levels and that transcription of the Csr sRNAs was dependent upon VarA recognition sequences in the promoter regions of the *csrB*, *csrC*, and *csrD* genes. We showed further that CsrA binds directly to the *varA* mRNA both *in vitro* and *in vivo* (Fig. 6). Taken together, these data indicate that CsrA interacts directly with the *varA* mRNA to ultimately increase expression of the Csr sRNAs. Our model of regulation, outlined in Fig. 1A, is as follows: when unsequestered CsrA exceeds a threshold, CsrA can bind to the *varA* transcript to facilitate translation. An increase in VarA leads to increased production of the Csr sRNAs, which bind to CsrA and reduce its availability. This feedback mechanism likely prevents drastic fluctuations in CsrA activity, ensuring optimal levels of active CsrA within the cell, and adds to the complex regulation of CsrA activity in V. cholerae.

## MATERIALS AND METHODS

### Bacterial strains and growth conditions.

All bacterial strains and plasmids used in this study are listed in Table S1 in the supplemental material. All strains were maintained at −80°C in tryptic soy broth–20% (vol/vol) glycerol. Cultures were grown at 37°C with shaking in Luria-Bertani (LB) broth (1% [wt/vol] tryptone, 0.5% [wt/vol] yeast extract, 1% [wt/vol] NaCl) or on LB agar plates. For the experiments, the strains were grown at 37°C with shaking in the minimal medium, namely, Tris-buffered minimal medium (T medium) (53), modified to contain 0.2% (wt/vol) sucrose instead of glucose and supplemented with VA vitamin solution (https://www.genome.wisc.edu/resources/protocols/ezmedium.htm). NRES supplement is a mixture of the amino acids asparagine, arginine, glutamate, and serine, each dissolved in water and supplemented at a final concentration of 3.125 mM. The following antibiotics were used at the indicated concentrations: for E. coli, 50 μg/ml kanamycin, 50 μg/ml ampicillin, 30 μg/ml chloramphenicol, and 250 μg/ml carbenicillin; for V. cholerae, 50 μg/ml kanamycin, 25 μg/ml ampicillin, 6 μg/ml chloramphenicol, 125 μg/ml carbenicillin, and 20 μg/ml polymyxin B. Plasmids were transferred into V. cholerae either by electroporation as previously described (54) or by conjugation with the help of MM294/pRK2013, which encodes the genes necessary for conjugation.

10.1128/mBio.01042-19.4 TABLE S1 Strains and plasmids. Download Table S1, DOCX file, 0.02 MB. Copyright © 2019 Butz et al. 2019 Butz et al. This content is distributed under the terms of the Creative Commons Attribution 4.0 International license.

### Construction of V. cholerae strains and plasmids.

Chromosomal deletions and fusion proteins were constructed by splice overlap PCR using the primers listed in Table S2. To perform the splice overlap, two 700-to-1,000-bp PCR products flanking the target of interest were generated. Upstream product A was generated by using primers annotated A.F. (product A forward) and A.R. (product A reverse). Downstream product B was generated using primers B.F. and B.R. These two flanking products were engineered to have overlapping sequences, enabling the products to anneal and generating product C, which was then amplified using primers annotated C.F. and C.R. To generate deletions, product C contained only the upstream and downstream flanking sequence of the gene of interest; the gene itself was not included. For fusion proteins, the epitope tag was included in the product A or product B sequence, as indicated in Table S2. In product C of fusion proteins, the stop codon of the protein of interest was changed to a glycine, immediately followed by additional glycine residues to serve as a linker between the protein of interest and one V5 epitope sequence (55). The product C fragments were cloned as a blunt-end fragment into the SmaI site of plasmid pCVD442N, generating pS*csrB*, pS*csrC*, pS*csrD*, and pS*varA*-V5. The allelic exchange constructs were transferred into N16961 and N*csrA*.R6H by conjugation, and allelic exchange was performed as described previously (56). To construct the strains containing a single Csr sRNA, two of the three genes were deleted by sequential allelic exchange. The sequences of all final products were verified at The University of Texas at Austin DNA sequencing facility using 3730/3730 XL DNA analyzers (Applied Biosystems).

10.1128/mBio.01042-19.5 TABLE S2 Primers. Download Table S2, DOCX file, 0.02 MB. Copyright © 2019 Butz et al. 2019 Butz et al. This content is distributed under the terms of the Creative Commons Attribution 4.0 International license.

To create the *csr* transcriptional fusions, the predicted full-length promoter (20 nucleotides upstream of the predicted VarA-binding site to +3 nucleotides relative to the transcriptional start site) was PCR amplified using primers listed in Table S2. The CsrB construct corresponded to the sequence extending from −197 to +3 relative to the start of transcription, the CsrC construct corresponded to the sequence extending from −196 to +3 relative to the start of transcription, and the CsrD construct corresponded to the sequence extending from −197 to +3 relative to the start of transcription. The truncated promoter fragments lacked 50 nucleotides at the 5′ end, including the putative VarA-binding site. The *varA* transcriptional reporter included 301 nucleotides upstream of the transcriptional start site and stopped 1 nucleotide before the transcriptional start site (−300 to −1 relative to the transcriptional start site). These PCR fragments were digested with NcoI and BamHI and cloned into the NcoI and BamHI sites located upstream of a promoterless *lacZ* gene in plasmid pQF50, generating the full-length reporter constructs pQFCsrBF1, pQFCsrCF1, and pQFCsrDF1 and the truncated reporter constructs pQFCsrBF2, pQFCsrCF2, and pQFCsrDF2, as well as the *varA* transcriptional reporter pQF*varA*.TS.

To generate the *varA* translational reporter, the translational reporter pQE*lacZ* was constructed first. The *lacZ* allele from E. coli (MM1655) was amplified with primers Ec.*lacZ*.F(SalI) and Ec.*lacZ*.R(PstI). These primers were engineered to generate a truncated *lacZ* gene product by initiating amplification at the 28th nucleotide. This product was then inserted into the SalI and PstI sites of pQE-2. The *varA* construct began 1 nucleotide upstream of the transcriptional start site, as determined by Papenfort et al. (57), and extended 220 nucleotides into the coding sequence (−1 to +312 relative to the transcriptional start site). This promoterless *varA* construct was cloned into the MfeI and NotI sites downstream of the T5 promoter in pQELacZ to generate translational reporter plasmid pQE*varA*.TL.

### RNA isolation and reverse transcriptase quantitative PCR (qRT-PCR) analysis.

LB cultures from single colonies were grown overnight and then diluted 1:100 in T medium with or without NRES and grown to the mid-log phase (optical density at 650 nm [OD_650_] of approximately 0.6), unless otherwise noted. To isolate total RNA, ∼10^9^ cells was added directly to RNA-Bee (Tel-Test, Inc.) and subsequently treated with DNase I (NEB) per the manufacturer’s directions. The ethanol-precipitated purified RNA pellets were resuspended in diethyl pyrocarbonate (DEPC)-treated water (Thermo Fisher). The RNA was reverse transcribed using a SuperScript III system (Thermo Fisher), and quantitative PCR (qPCR) was performed with Power SYBR green (Thermo Fisher). All of these analyses used *secA* as an internal reference, unless otherwise specified. An Applied Biosystems ViiA 7 instrument was used for qPCR with the following parameters: for the holding stage, 50°C for 2 min and 95°C for 10 min; for the PCR stage, 95°C for 15 s and 60°C for 1 min repeated 40 times, with the fluorescence recorded at 60°C; for the melting curve stage, 90°C for 15 s, 60°C for 1 min, and then 95°C for 15 s with the fluorescence recorded every 0.05 s. Standard curves generated from purified *csrB*, *csrC*, *csrD*, and *secA* PCR products were used to determine the concentrations via the use of QuantStudio Real-Time software v1.3. Relative levels were calculated using the threshold cycle (ΔΔ*C_T_*) method. Each reaction produced only one melting curve, indicating that only one target had been amplified during the qPCR reaction.

### SDS-PAGE and immunoblotting.

Overnight cultures from a single colony were diluted 1:100 into T medium with or without NRES. Cultures were grown to mid-log phase (OD_650_ of approximately 0.6), and samples containing an equivalent number of cells were resuspended in Laemmli SDS sample buffer (58). Samples were resolved by electrophoresis through 10% SDS-polyacrylamide gels, and the proteins were visualized by Coomassie brilliant blue staining or immunoblotting. On the Coomassie gels, the positions of the OmpT and OmpU proteins were determined by comparisons with previous studies of the wild-type strain performed with *ompT* and *ompU* mutant strains (59). The proteins for immunoblotting were transferred to a 0.45 μm-pore-size nitrocellulose membrane (GE Healthcare). ToxR was detected using rabbit polyclonal anti-ToxR antiserum (a gift from R. K. Taylor) (diluted 1:1,000). V5 fusion proteins were immunodetected by the use of mouse monoclonal anti-V5 antibodies (Sigma-Aldrich; catalog no. V8012) (diluted 1:10,000). To visualize the proteins, either horseradish peroxidase (HRP)-conjugated goat anti-rabbit IgG or HRP-conjugated goat anti-mouse IgG (Bio-Rad Laboratories) (diluted 1:10,000) was used. To ensure equal levels of loading, the relative densities of the immunoblotted samples were assessed by Coomassie staining of a duplicate gel.

### LacZ fusion reporter assay.

The transcriptional (pQF*varA*.TS) and translational (pQE*varA*.TL) reporters were electroporated into the *lacZ* mutant strains (N*lacZ*::*kan* and N*csrA*.R6H.*lacZ*::*kan*). The transformed strains were grown to an OD_650_ of approximately 0.6 in minimal medium supplemented with NRES. Levels of β-galactosidase activity were measured as described previously by Miller (60).

### RNA stability.

Overnight cultures from a single colony were diluted 1:100 into T medium plus NRES. Cultures were grown to mid-log phase (OD_650_ of approximately 0.6), and 200 μg/ml of rifampin was added to the culture to stop transcription. Cells were harvested immediately (*t* = 0) and at 4, 8, 16, and 64 min following the rifampin addition and were added directly to RNA stabilizing solution (5% phenol, 95% ethanol). RNA was then purified from the samples as described above and used to generate cDNA. The Csr RNA species, along with control 16S rRNA, were detected by qRT-PCR. The percentage of each of the Csr sRNAs remaining at each time point was calculated relative to *t* = 0 (100%). The half-lives were determined using one-phase decay analysis (a nonlinear regression method) and GraphPad Prism 7 software. To perform statistical analyses, the extra sum-of-squares F-test was used to compare the rates of decay.

### CsrA purification.

Strain BL21DE3/pET16b-His-CsrA, a generous gift from Charles Midgett, was grown to an OD_650_ of approximately 0.6 at 37°C with shaking. CsrA production was induced by the addition of 500 μM IPTG (isopropyl-β-d-thiogalactopyranoside) for 4 h at 23°C with shaking. The cells were centrifuged at 5,500 × *g* for 10 min at 4°C, washed with 50 mM NaCl, and stored at −80°C. The cells were lysed by resuspending the cells in 10 ml of lysis buffer (50 mM NaH_2_PO_4_, 200 mM NaCl, 10 mM imidazole, 1% Triton X-100, 10% glycerol, and one tablet of cOmplete, Mini, EDTA-free protease inhibitor cocktail (Sigma-Aldrich) followed by four rounds of 30 s of sonication, each followed by 30 s on ice. Cell debris was pelleted by centrifugation at 15,000 × *g* at 4°C. The supernatant was incubated with 5 ml of nickel agarose beads for 3 h with rotation. The slurry was washed with 50 ml of wash buffer (50 mM NaH_2_PO_4_, 300 mM NaCl, 20 mM imidazole, and 0.1% Triton X-100). Elution buffer (50 mM NaH_2_PO_4_, 300 mM NaCl, 500 mM imidazole) was used to elute His-TEV-CsrA in 1-ml fractions. Relevant factions were pooled, tobacco etch virus (TEV) protease was added at a 1:10 (TEV/CsrA) ratio, and the reaction mixture was incubated overnight at 4°C. The reaction mixture was then passed through a nickel agarose column, and purified CsrA was concentrated and stored in storage buffer (50 mM KPO_4_, 50 mM NaCl, 50% glycerol).

### RNA electrophoretic mobility shift assays (RNA EMSA).

The RNA oligonucleotides, listed in Table S2, were produced by IDT Technologies and were designed to have a single biotin molecule on the 5′ end. The CsrA-RNA EMSA samples were prepared using a LightShift chemiluminescent RNA EMSA kit according to the instructions of the manufacturer (Thermo Fisher), with the slight modification of setting the final volume to equal 10 μl instead of 25 μl. CsrA-RNA complexes were resolved by electrophoresis through a 12% nondenaturing polyacrylamide gel, transferred to a BrightStar-Plus (Thermo Fisher) positively charged nylon membrane, and subjected to UV cross-linking (150 mJ). A LightShift chemiluminescent RNA EMSA kit was used to visualize the biotinylated RNA.

### CsrA-VarA coimmunoprecipitation.

DNA corresponding to *csrA* tagged with a single V5 tag sequence on the C terminus inserted into the inducible vector pBAD18-cm was generously provided by Bryan Davies (University of Texas at Austin). CsrA-V5 expression was induced in mid-log phase culture by adding 0.1% arabinose to the medium for 15 min. The cells were centrifuged and resuspended in 2.5 ml of binding/wash buffer (100 mM MOPS [morpholinepropanesulfonic acid] [pH 7.0] [KOH], 10 mM MgCl_2_, 100 mM KCl). The cells were lysed in a French pressure cell, with a final volume of ∼2 ml. A 100-μl volume of the cell lysate was added directly to RNA-Bee, and the RNA was further purified. The remaining lysate was incubated with protein G Dynabeads (Thermo Fisher) coated with anti-V5 antibodies (Sigma-Aldrich; catalog no. V8012). The beads were washed with binding/wash buffer. The beads were resuspended in 100 μl of buffer and added to RNA-Bee before further purification. CsrA binding to *varA* mRNA was determined via qRT-PCR by comparing the relative abundances of *varA*, normalized to the internal control *secA* level, in the immunoprecipitated RNA and in the input RNA.
