# Supplementary material for: Vibrio cholerae CsrA Directly Regulates varA To Increase Expression of the Three Nonredundant Csr Small RNAs
Source: mBio. 2019 Jun 4;10(3):e01042-19. doi: 10.1128/mBio.01042-19 (PMC6550530; doi:10.1128/mBio.01042-19)
Supplement: TABLE S1 [file mBio.01042-19-st001.docx]

Supplemental Table 1

Table S1. Strains and Plasmids

| **Strains** | **Description** | **Source or reference** |
| --- | --- | --- |
| N16961 | Wild-type *V. cholerae* El Tor biotype | R. A. Finkelstein |
| N*csrA*.R6H | N16961 *csrA*.R6H | (1) |
| N*csrB-only* | N16961 *∆csrC ∆csrD* | This study |
| N*csrC-only* | N16961 *∆csrB ∆csrD* | This study |
| N*csrD-only* | N16961 *∆csrB ∆csrC* | This study |
| N*lacZ::kan* | N16961 *∆* *lacZ*::kan | This study |
| N*csrA.R6H.lacZ::kan* | N*csrA*.R6H *∆* *lacZ*::kan | This study |
| N*varA-V5* | N16961 *varA*-V5 | This study |
| N*csrA.R6H.varA-V5* | N*csrA*.R6H *varA*-v5 | This study |
| DH5α *(λpir)* | *Escherichia coli* cloning strain | J. Kaper |
| MM294/pRK2013 | *Escherichia coli* conjugation helper strain | R. Meyer |
| BL21DE3/pET16b-His CsrA | Purification of *V. cholerae* CsrA | Charles Midgett |
| **Plasmids** |  | **Reference** |
| pCC1 | Single-copy no. cloning vector; cam^R^ | Epicenter |
| pCVD442N | Suicide vector pG704 carrying sacB; amp^R^, Suc^s^ | (2) |
| pQF50 | Promoterless *lacZ* reporter plasmid; amp^R^ | (3) |
| pQE-2 | IPTG-inducible expression vector | Qiagen |
| pF*csrA* | pCC1 carrying csrA; cam^R^ | (1) |
| pF*csrA-V5* | pCC1 carrying *csrA*-V5; cam^R^ | This study |
| pBAD*csrA-V5* | pBAD18-cm carrying csrA-V5 with a 2- glycine linker; cam^R^ | Bryan Davies |
| pF*csrA-V5(5G)* | pCC1 carrying *csrA*-V5 with a five- glycine linker; cam^R^ | This study |
| pS*csrB* | pCVD442N carrying a construct to delete CsrB; amp^R^ | This study |
| pS*csrC* | pCVD442N carrying a construct to delete CsrC; amp^R^ | This study |
| pS*csrD* | pCVD442N carrying a construct to delete CsrD; amp^R^ | This study |
| pS*lacZ::kan* | pCVD442N carrying a construct to replace *lacZ* with a kanamycin cassette; amp^R^ | Stephanie A. Craig |
| pS*varA-V5* | pCVD442N carrying a construct to chromosomally V5 epitope tag the C-terminus of VarA with a 2 glycine linker; amp^R^ | This study |
| pQFCsrB.F1 | pQF50 carrying the full-length *csrB* promoter; amp^R^ | This study |
| pQFCsrB.F2 | pQF50 carrying the truncated *csrB* promoter; amp^R^ | This study |
| pQFCsrC.F1 | pQF50 carrying the full-length *csrC* promoter; amp^R^ | This study |
| pQFCsrC.F2 | pQF50 carrying the truncated *csrC* promoter; amp^R^ | This study |
| pQFCsrD.F1 | pQF50 carrying the full-length *csrD* promoter; amp^R^ | This study |
| pQFCsrD.F2 | pQF50 carrying the truncated *csrD* promoter; amp^R^ | This study |
| pQF*varA*.TS | pQF50 carrying the *varA* promoter; amp^R^ | This study |
| pQE*lacZ* | pQE2 carrying a *lacZ* allele with the first 9 amino acids not included; amp^R^ | This study |
| pQE*varA*.TL | pQELacZ carrying the *varA* translational fusion to lacZ; amp^R^ | This study |

1. Mey AR, Butz HA, Payne SM. *Vibrio cholerae* CsrA regulates ToxR levels in response to amino acids and is essential for virulence. mBio. 2015 Sep 1;6(4):e01064-15.

2. Wyckoff EE, Mey AR, Leimbach A, Fisher CF, Payne SM. Characterization of ferric and ferrous iron transport systems in *Vibrio cholerae*. J Bacteriol. 2006 Sep;188(18):6515–23.

3. Farinha MA, Kropinski AM. Construction of broad-host-range plasmid vectors for easy visible selection and analysis of promoters. J Bacteriol. 1990 Jun;172(6):3496–9.
