# Supplementary material for: Vibrio cholerae CsrA Directly Regulates varA To Increase Expression of the Three Nonredundant Csr Small RNAs
Source: mBio. 2019 Jun 4;10(3):e01042-19. doi: 10.1128/mBio.01042-19 (PMC6550530; doi:10.1128/mBio.01042-19)
Supplement: TABLE S2 [file mBio.01042-19-st002.docx]

Supplemental Table 2

Table S2. Primers

| Purpose | Oligo name | 5' to 3' |
| --- | --- | --- |
| Transcript-ional reporters | CsrB.ts.F | ATGACCATGGCCGACTTTGGCCGATCAT |
|  | CsrB.ts.F2 | ATGACCATGGAAATTCGCTTTCTGCGAT |
|  | CsrB.ts.R | ATGAGGATCCGACGAGATTCATCTTACTG |
|  | CsrC.ts.F | ATGACCATGGGAGCAAAAGGGGCAATCG |
|  | CsrC.ts.F2 | ATGACCATGGAAAAACAGTGAATTGATGC |
|  | CsrC.ts.R | ATGAGGATCCCGACAAGAGATAATTTACC |
|  | CsrD.ts.F | ATGACCATGGTCTTATTTTAGGATTTATCT |
|  | CsrD.ts.F2 | ATGACCATGGGATTTTAGCTTTTGATTGC |
|  | CsrD.ts.R | ATGAGGATCCGACGAATTGACTTTACG |
|  | VarA.ts.F | ATGCCCATGGCCTTGTTCAATCTTCAATCTGG |
|  | VarA.ts.R | GATCGGATCCCCCGTGAATATGATCTGAAACC |
| Translational reporters | Ec.lacZ.F(SalI) | TTgGTCGACGTCGTTTTACAACGTCGTG |
|  | Ec.lacZ.R(PstI) | TTGCTGCAGTTATTTTTGACACCAGACCAACTGG |
|  | VarA.TL.F1 | ATGACAATTGAGAAAAGTTGCCCGCGTTGG |
|  | VarA.TL.R1 | ATGCGCGGCCGCGATTAACACGCAACAGCTT |
| V5-tagging VarA | VarA.F (A.F., C.F.) | TCATAATCACTCGGCTGTCCATCGGG |
|  | VarA-V5.R (A.R.) | **AGGAGAGGGTTAGGGATAGGCTTACC**ACCACCTAACTTCTCAGTATCTAAGATCCC |
|  | VarA-V5.F (B.F.) | **GGTAAGCCTATCCCTAACCCTCTCCTCGGTCTCGATTCTACGTAA**TGTCGACACAGTTTGATTCTGCCCC |
|  | VarA.R (B.R., C.R) | CCTCGATTTAAAATGATCCGTGCCGG |
| RNA EMSA probes | varA | AAGUGUGGAGAUACAAGUUUGAUUAGUGUUUUCCUUGUAGAUGAUCACGAGCUGGUUCGCACAGGGAUACGACGUAUUAUUGAAGACGUCCGUGGAAUGAAAGUAGCAGGGGAAGCUGAC |
|  | atpI  (negative control) | AAAAUUGUAACUUUUUGCGGUGUUUUCAUUGCAAUCAGUUGAUGCCACCGUAUAAUUUUCGCCAAUUUCCCAGUCCGAAAAAGCGGUUCGAGCUGUGGUGAUUACUAGAGGUAAGAAUAC |
| CsrB deletion | csrB.F (A.F.) | CGCTTGGTTACGACGGCTACGC |
|  | csrB.F3 (C.F.) | AGATGTCGCGTATCAGGTCG |
|  | csrB.del1 (A.R.) | CGGGAGCCCGGGAGATTCATCTTACTGAGCTGAGC |
|  | csrB.del2 (B.F.) | AATCTCCCGGGCTCCCGAAAAGTCATCACC |
|  | csrB.R (B.R.) | TTGGCCTACCGCATTTATGGCG |
|  | csrB.R3 (C.R.) | TGTCGGTCTGCTCAATGACC |
| CsrC deletion | csrC.F (A.F., C.F.) | CGCCACTTACTACACGCCATAGTG |
|  | csrC.del1 (A.R.) | AGGAAAGCCCGGGAAGAGATAATTTACCC |
|  | csrC.del2 (B.F.) | TATCTCTTCCCGGGCTTTCCTTAATCTCCC |
|  | csrC.R (B.R., C.R.) | GAGTGATGCTGCCTCTATTGCAC |
| CsrD deletion | csrD.F (A.F.) | ACGATGGTTGCTGGTTATCCC |
|  | csrD.F3 (C.F) | GGCGATCTTTGTCATCTGCG |
|  | csrD.del1 (A.R.) | AAGATGCCCGGGAATTGACTTTACGATATGAGCG |
|  | csrD.del2 (B.F.) | AGTCAATTCCCGGGCATCTTTCCGTATGCC |
|  | csrD.R (B.R.) | CTGGTGGCAATTCGCTCTGGC |
|  | csrD.R3 (C.R.) | CGCTTAGGCATCAAATCCGC |
| qPCR | csrB.F | GGACATTGAACGGACGCAATCG |
|  | csrB.R | TGATAGTACGCTGCAATCCG |
|  | csrC.F | AGGTGAGCTGCAAGGATGAT |
|  | csrC.R | CTTGTTCCTTGAGCGGTGTC |
|  | csrD.F | CAGGATGAATGGTCGGCAAG |
|  | csrD.R | CCTCTGCCATCCTGACAGAA |
|  | secA.F | GCGTACGGGTGAAGGTAAAA |
|  | secA.R | CCAGGTAGGGCATTCAGGTA |
|  | 16s | CAGCCACACTGGAACTGAGA |
|  | 16s | TTAGCCGGTGCTTCTTCTGT |
| Csr specific RT probe | Csr.RT | TCGCAGCA |
| Standard curve | CsrB.F1 | ATGCTCGGCAAGGAAAGCG |
|  | CsrB.R1 | CCAGCTACTAAAAGGTGCTCCC |
|  | CsrC.F1 | ATGACCACAAGCAGGAAGCC |
|  | CsrC.R1 | CTCCATGCATCATCCTTAACGGG |
|  | CsrD.F1 | GGAAGCGGACACGGAACAGG |
|  | CsrD.R1 | TCTCACTCGCAGCAATCCCG |
|  | SecA.F1 | GTCAACGCATTGAACAGGGC |
|  | secA.R1 | TTCACACCCACGGTCATGCC |

Restriction enzyme recognition sequences are underlined.

Sequence encoding the V5-epitope is bolded**.**
